# Modelling the Seasonal Epidemics of Respiratory Syncytial Virus in Young Children

**DOI:** 10.1371/journal.pone.0100422

**Published:** 2014-06-26

**Authors:** Hannah C. Moore, Peter Jacoby, Alexandra B. Hogan, Christopher C. Blyth, Geoffry N. Mercer

**Affiliations:** 1 Telethon Kids Institute, University of Western Australia, Perth, Australia; 2 National Centre for Epidemiology and Population Health, Australian National University, Canberra, Australia; 3 School of Paediatrics and Child Health, University of Western Australia, Perth, Australia; 4 Department of Paediatric and Adolescent Medicine, Princess Margaret Hospital for Children, Perth, Australia; Nanyang Technical University, United States of America

## Abstract

**Background:**

Respiratory syncytial virus (RSV) is a major cause of paediatric morbidity. Mathematical models can be used to characterise annual RSV seasonal epidemics and are a valuable tool to assess the impact of future vaccines.

**Objectives:**

Construct a mathematical model of seasonal epidemics of RSV and by fitting to a population-level RSV dataset, obtain a better understanding of RSV transmission dynamics.

**Methods:**

We obtained an extensive dataset of weekly RSV testing data in children aged less than 2 years, 2000–2005, for a birth cohort of 245,249 children through linkage of laboratory and birth record datasets. We constructed a seasonally forced compartmental age-structured Susceptible-Exposed-Infectious-Recovered-Susceptible (SEIRS) mathematical model to fit to the seasonal curves of positive RSV detections using the Nelder-Mead method.

**Results:**

From 15,830 specimens, 3,394 were positive for RSV. RSV detections exhibited a distinct biennial seasonal pattern with alternating sized peaks in winter months. Our SEIRS model accurately mimicked the observed data with alternating sized peaks using disease parameter values that remained constant across the 6 years of data. Variations in the duration of immunity and recovery periods were explored. The best fit to the data minimising the residual sum of errors was a model using estimates based on previous models in the literature for the infectious period and a slightly lower estimate for the immunity period.

**Conclusions:**

Our age-structured model based on routinely collected population laboratory data accurately captures the observed seasonal epidemic curves. The compartmental SEIRS model, based on several assumptions, now provides a validated base model. Ranges for the disease parameters in the model that could replicate the patterns in the data were identified. Areas for future model developments include fitting climatic variables to the seasonal parameter, allowing parameters to vary according to age and implementing a newborn vaccination program to predict the effect on RSV incidence.

## Introduction

Respiratory syncytial virus (RSV) is the most common cause of acute lower respiratory infections (ALRI) in children, especially those aged less than two years. While RSV is not a notifiable disease, the health and economic burden for young children surpasses that of influenza in Australia, with an estimated incidence ranging from 435–869/1000 infant population [1]. In the United Kingdom RSV mortality rates in those aged less than 15 years have been shown to be on par with those of influenza [2]. RSV follows a distinct seasonality in most geographical areas with peaks during winter months associated with cooler temperatures and increased rainfall, [3]–[6] however RSV incidence can be highly variable within countries and between regions within a country depending on the level of seasonal forcing [7].

Mathematical models have been used in epidemiology to characterise annual epidemics of respiratory infections and measure the likely impact of intervention programs [8], [9]. Models can also determine the optimum timing of interventions and the proportion of the population that need to be vaccinated in order to reach the herd immunity threshold. There is currently no licenced vaccine for RSV, but immunoprophylaxis with RSV-specific monoclonal antibodies is effective in reducing RSV-related severe disease [10]. Furthermore, an attenuated intranasal RSV/parainfluenza virus type 3 vaccine (MEDI-534) and a live attenuated intranasal RSV vaccine (MEDI-559) have recently undergone Phase 1 clinical trials in infants and young children [11], [12]. Mathematical models are likely to be useful in assessing the impact of such vaccines. However, to improve the accuracy of these models, real population data are needed to fit the model parameters. Unlike notifiable diseases where positive detections are reported nationally, RSV models rely on hospitalisations or routinely collected laboratory data. To date, variations of simple compartmental models for RSV have been constructed using hospitalisation data from The Gambia, Singapore, Florida (USA) and Finland [13] and Spain [14]. A further model has since been developed using RSV testing data from a major children’s hospital in Utah (USA) [15]. These models have shown that they can reliably mimic the distinctive seasonal characteristics of RSV. However, due to the differences in climate between these regions, which result in different levels of seasonal forcing, RSV models need to be developed and fitted to specific geographical areas.

We have access to total population-based linked data including state-wide routinely collected laboratory data through the Western Australian Data Linkage System (WADLS). Demographic, clinical and laboratory data are linked following a best practice protocol [16] in which personal identifiers are separated from health and laboratory data and linked by a separate linkage team to produce a project-specific child identifier key. De-identified data for each of the datasets are then given to the approved research team for analysis. In this study, we establish a compartmental model of RSV transmission based on positive detections of RSV in metropolitan Western Australian children, obtained through population-based data linkage. Secondly, we explore variations in the model fit with changes in the disease parameters of the latency and infectious periods, and the reduction in the susceptibility and infectiousness for older children and adults due to reinfection and ageing.

## Methods

### Population and Setting

Western Australia covers approximately 2.5 million square kilometres and has a population of 2.2 million [17]. The Western Australian Department of Health classifies residential postcodes into three major geographical regions: metropolitan (the capital city, Perth, and its surrounds), rural (South West and North Eastern areas outside of the metropolitan region) and remote (the far East and far North Western Australia). The state crosses several climatic zones with a temperate climate in the metropolitan and southern regions and a tropical climate in the northern regions. Approximately three-quarters of Western Australia’s population reside in the metropolitan area. There is one dedicated tertiary level paediatric teaching hospital, Princess Margaret Hospital for Children (PMH) located in the state capital, Perth. At PMH, it is standard practice to collect nasopharyngeal aspirates (NPA) for respiratory virus detection on all children admitted to hospital with ALRI [18]. A recommendation for respiratory pathogen testing is in place at other smaller metropolitan and non-metropolitan hospitals across Western Australia.

### Population-based data

Previously we have extracted data from WADLS on 245, 249 singleton live births (7.1% of which are Aboriginal) in Western Australia between 1996 and 2005 from the Midwives’ Notification System, Birth and Death Register and the Hospital Morbidity Database System. Details of data cleaning are provided elsewhere [19], [20]. In brief, our linked dataset contains information on birth details, demographics and hospitalisation episodes for ALRI between 1996 and 2005.

We have also extracted data from the PathWest Laboratory Database concerning routine detections of respiratory viruses and bacteria. PathWest Laboratory Medicine Western Australia (PathWest) is the government-funded public laboratory service and consists of all public pathology laboratories in Western Australia and carries out a full range of diagnostic testing for infectious diseases. Details of the component datasets within the PathWest Laboratory Database are given elsewhere [21]. Laboratory data were available from 2000 to 2005 for all children in the birth cohort for ages 0–9 years. Respiratory samples received at PathWest for viral testing are routinely investigated for RSV, influenza viruses A and B, adenoviruses and parainfluenza virus types 1–3. RSV was detected by either immunofluorescent antigen detection, polymerase chain reaction (PCR) and/or viral culture. Testing methods for RSV did not change between 2000 and 2005.

We used the residential postcode of the mother at the time of her child’s birth as recorded on the Midwives’ Notification System to classify laboratory detections to either the metropolitan, rural and remote regions of Western Australia. To establish a model of metropolitan RSV transmission in a temperate climate, we restricted our data to RSV testing and positive detections in children born in metropolitan areas. Ethical approval for this study was granted by Princess Margaret Hospital for Children Ethics Committee and the Department of Health Western Australia Human Research Ethics Committee. Access to the population-based data was approved by the Western Australian Data Linkage Branch.

### Model structure

**Figure 1:**
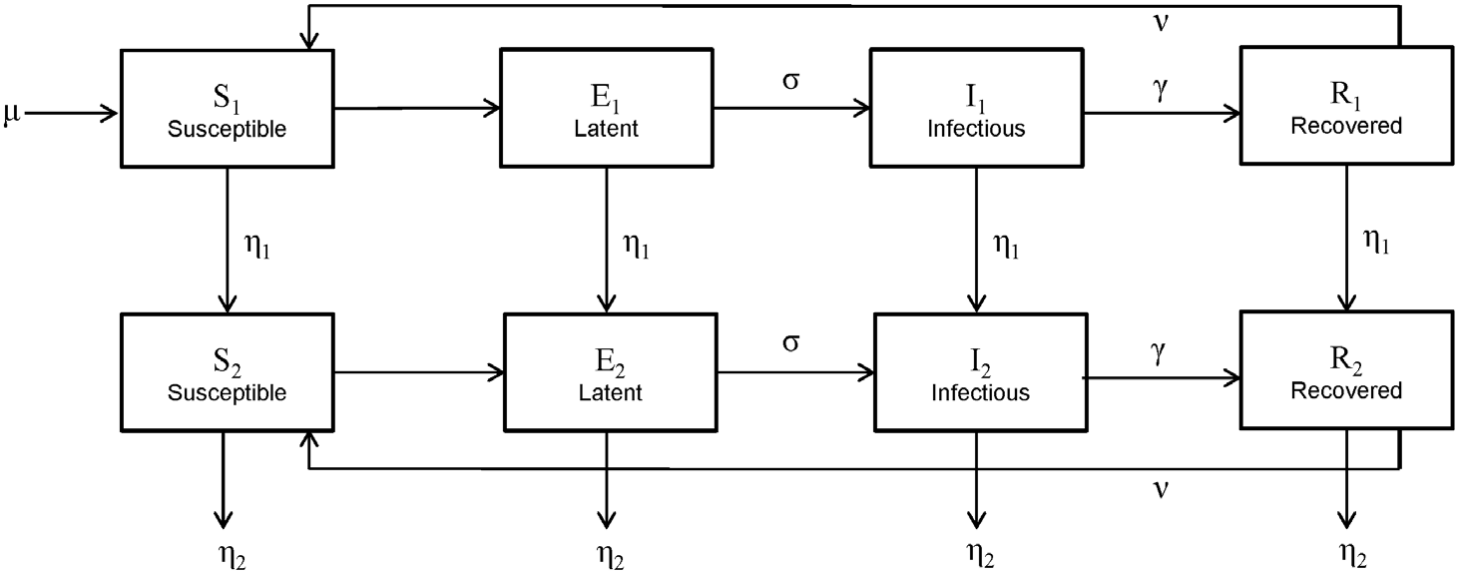
Schematic representation of RSV SEIRS model with two age groups. (SEIRS)_1_ represents those aged less than 2 years and (SEIRS)_2_ represents those aged 2 years or more.

We adapted the RSV compartmental transmission models used by others [13], [15], [22] and constructed a Susceptible (S) – Exposed (E) – Infectious (I) – Recovered (R) – Susceptible (S) model with two age classes. S_1_, E_1_, I_1_ and R_1_ represented those individuals aged less than 2 years and S_2_, E_2_, I_2_, and R_2_ represented those individuals aged 2 years and over. Our SEIRS model was defined by a series of differential equations:
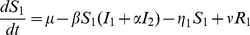


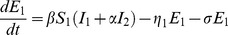


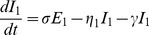


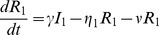


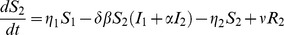


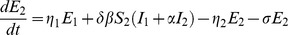


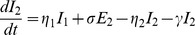


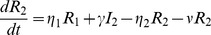
where: µ is the birth rate, β is the disease transmission rate, η_1_ is the ageing rate from (SEIR)_1_ to (SEIR)_2_, η_2_ is the ageing rate of leaving (SEIR)_2_ which is related to birth/death rate, 1/σ is the average latency period, 1/γ is the average infectious period and 1/ν is the average duration of immunity. The scaling parameters α and δ represent the reductions in infectivity and susceptibility respectively for the older age group compared to those aged less than 2 years. Each model class represents the proportion of the total population. A schematic representation of the model is shown in Figure 1.

### Parameters

The parameters were based either on real data from our previous population-based data linkage studies, estimates from the literature or were estimated during the model fitting process. Data were aggregated at the weekly level so the time period of interest is a week and all rates are quoted as per week for consistency.

#### Birth rate (µ) and ageing rates (η1 and η2)

Age class 1 represented those aged less than 2 years and age class 2 represented those aged 2 years or more. Therefore, the weekly ageing rate was assumed to be 1/(2*52) for moving from (SEIR)_1_ to (SEIR)_2_. We assumed an overall stable population with a life expectancy of 80 years and that deaths in those aged less than 2 years are insignificant to the older age group following Keeling and Rohani [22]. Therefore the rate of entering the model, or the birth/death rate, was assumed to be equal to the ageing rate for leaving (SEIR)_2_ and was calculated as 1/(78*52).

#### Latency period (1/σ)

The latency period is defined as the time between being infected and becoming infectious. Based on previous literature [13], this was initially assumed to be an average of 4 days (0.57 of a week). Values between 2 and 6 days were later tested to determine the effect on the model fit.

#### Infectious period (1/γ)

The infectious period estimates the time an individual remains infectious. Previous RSV models have assumed this to be 10 days [13]–[15]. This estimate has been based on a previous analysis of 23 infants where the average duration of shedding of RSV was 6.7 days with a range from 1–21 days [23]. In order to be consistent with the literature, our initial model assumed an average infectious period of 10 days (1.4 weeks). Values between 7 and 12 days for this parameter were later tested to determine the effect on the model fit.

#### Duration of immunity (1/ν)

The duration of immunity is defined as the time between entering the recovery state to becoming susceptible again for a subsequent RSV infection. In previous literature [13] the duration of immunity has been estimated to be 200 days (25.6 weeks). In a more recent RSV model, the duration of immunity that produced the best fit to the data was a yearly rate of 1.59 which approximately translates to 230 days (32.9 weeks) [14]. Due to the seasonal nature of RSV epidemics the duration of immunity is difficult to measure from observational data as the risk of reinfection depends on the time of year of the first infection. An infection late in an epidemic season has a lower risk of reinfection that season than an earlier infection and is more likely to be reinfected in the following season. This effect greatly biases the calculation of the duration of immunity and probably leads to an overestimate. Due to the great uncertainty in this parameter and its impact on the outcome in the model we elected to make the duration of immunity a variable to be fitted rather than estimated from the literature.

#### Transmission parameter (β)

To account for the distinct seasonality of RSV, the disease transmission parameter was calculated as follows:
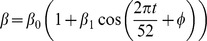



This is based on harmonic analysis that we have previously shown to accurately model RSV seasonality in metropolitan Western Australia [3]. This seasonality function allowing for varying amplitude over an annual cycle with a horizontal shift is common in modelling seasonal outbreaks and has been used in previous RSV transmission models [13], [14], [22]. The component parameters β_0_, β_1_ and φ (phase shift) were estimated through the model fitting process with *t* representing time in weeks. Here β_0_ is the average transmission rate and β_1_ is the degree of seasonality which is defined over the range [0,1] with higher values more appropriate for regions with stronger seasonal drivers.

#### Scaled susceptibility (δ)

To account for less reported infections in those aged 2 years or more, we included a scaling parameter to reduce the susceptibility of the older age class being infected by both infectious age classes. Based on reinfection studies [24], [25] initially the scaling parameter was set to 0.65 and later a range from 0.5 to 0.8 was considered.

#### Scaled infectiousness (α)

To account for reduced transmission in those aged 2 years or more, we included a scaling parameter to reduce the transmission from the infected population in the older age class, to the susceptible population in both age classes. The literature on the level of transmission from older children and adults to younger children is scarce, thus as a starting point in the model we set the scaled infectiousness to be the same as the scaled susceptibility, at 0.65 with a range from 0.5 to 0.8 considered.

### Model fitting

The SEIRS model was developed in Cran R statistical package [26], using the *deSolve* function to numerically solve the differential equations and verified in MATLAB. The model was used to fit the components of the seasonal transmission parameter, β_0_, β_1_ and φ, and the inverse of the immunity period ν. The model was fitted to data regarding positive RSV detections in children aged less than 2 years between 2000 and 2005 in metropolitan Western Australia. Since the model is a population wide model based on proportions in each class and the data are for reported RSV cases from population-based linked data, the model results were scaled to represent this proportion of the total community cases. This scaling was done by ensuring the total number of cases over the 6 years of the study and the model agreed. After using a set of initial conditions for β_0_, β_1_, φ and ν, we used the Nelder-Mead method in Cran R with the *neldermead* package [27] to fit the model to our actual RSV data. The weekly cumulative number of cases in the first age class (children aged less than 2 years) from the model and the real data was used in the model fitting process. The fit statistic that was minimised during the model fitting process was defined as:
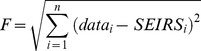
where data*_i_* and SEIRS*_i_* are the weekly number of cases aged less than 2 years in the data and SEIRS model outcome and *i* is the index for all weeks from the beginning of 2000 until the end of 2005. In contrast to Leecaster and colleagues [15] the fit statistic is not scaled by the model value as this gives a poorer fit due to the long periods over the summer months with very small case numbers.

In addition to fitting the components of the transmission parameter and the immunity parameter, we explored the variation in the fitted value of these four parameters and the model fit statistic by varying the duration of the latency period (2 to 6 days), the recovery period (7 to 12 days), and the scaled infectiousness and susceptibility parameters (0.5 to 0.8). The variations used were based on a literature search and the epidemiology of RSV detections and hospitalisations using our Western Australian data.

## Results

### RSV detections

**Figure 2:**
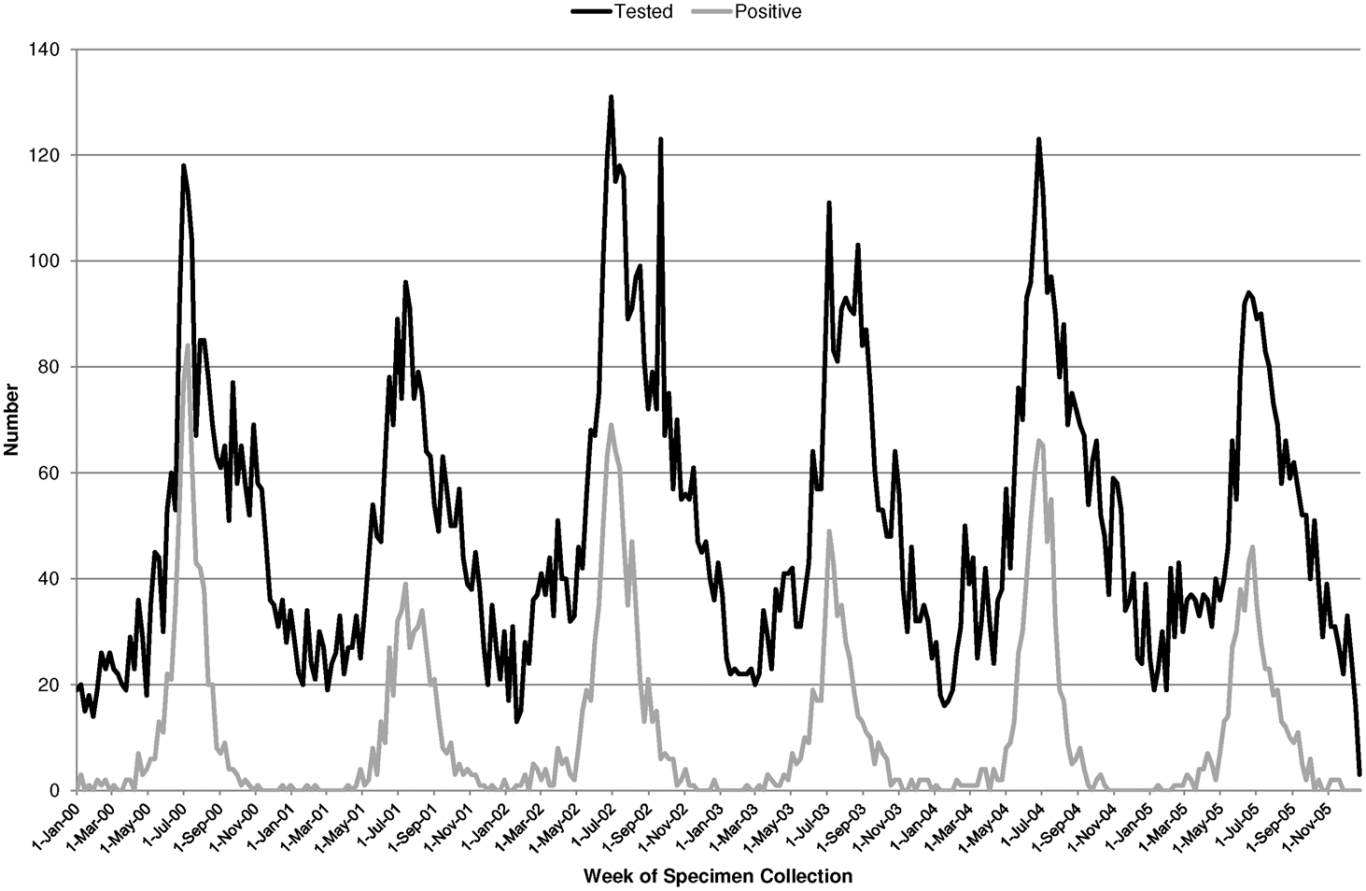
Weekly number of specimens tested and found positive for RSV in metropolitan Western Australian children aged less than 2 years from January 2000 to December 2005.

There were 15,830 specimens collected in metropolitan children aged less than 2 years between 2000 and 2005 that were tested for RSV with 21% (n = 3394) being positive for RSV by direct immunofluorescence, viral culture or PCR. Of the positive RSV detections, 89.7% were identified from specimens collected from hospitalised children, 9.4% were identified from specimens collected from non-hospital patients, that is patients attending emergency departments or hospital associated out-patient clinics, and the remaining 0.9% were collected in children from private laboratories associated with general practices. There were 4813 specimens in those aged 2–8 years that were tested and from those 11% (n = 534) were positive for RSV. Specimens tested and found positive for RSV followed a clear seasonal pattern with peaks in the winter months of June to August and very few cases during the summer months of December to February (Figure 2). There was also a distinct biennial seasonal pattern in both testing and positive detections with alternating peaks. The average maximum number of weekly detections in the higher peaks for years 2000, 2002 and 2004 was 73 compared with an average of 45 detections per week for years 2001, 2003 and 2005. The proportion of specimens tested that were positive for RSV ranged from 0% in off season months to 74% in the peak periods.

### SEIRS model

**Figure 3:**
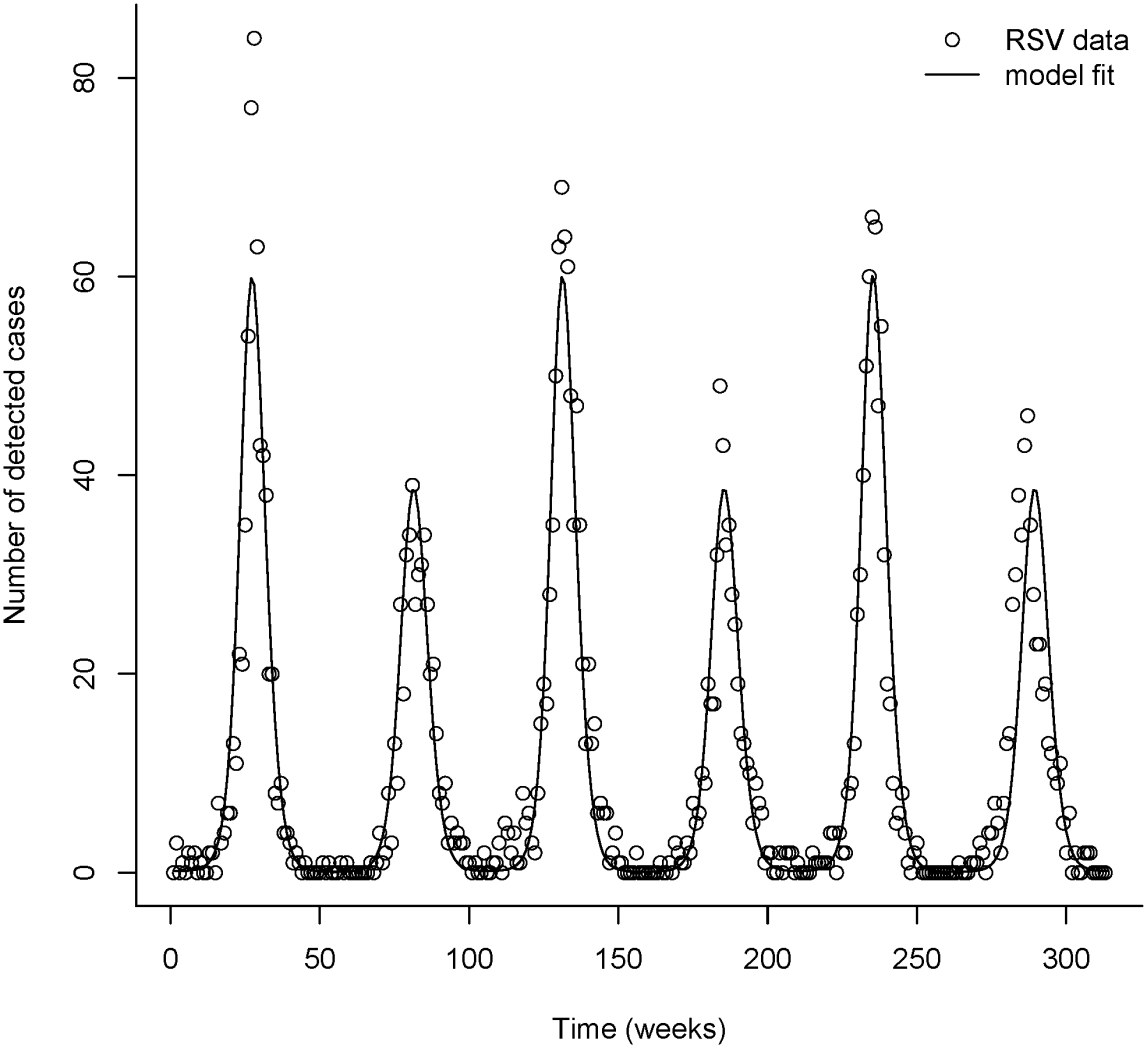
Observed RSV identifications in those aged less than 2 years and the fitted SEIRS model. The parameters used in the model are α = 0.65; δ = 0.65; **γ** = 1/1.4; and σ = 1/0.57, producing a fit statistic of F = 89.5751.

The SEIRS model was fitted to the 6 years of RSV positive identifications. The transmission parameter values that minimised the fit statistic using the Nelder-Mead method were β_0_ = 1.99, β_1_ = 0.65, φ = 2.43 and ν = 0.044 (translating to an immunity period of about 23.5 weeks), resulting in a fit statistic of F = 89.575. This best fit of the model to the data displayed alternating peak sizes that accurately mimicked the observed data (Figure 3). In contrast to the models of Leecaster and colleagues [15] the parameter values used here are constant across the entire dataset and do not vary by epidemic year. That is, there is a natural period 2 oscillation in the model with these parameter values giving the alternating peaks observed in the data. Using different parameter values for different years was not required to achieve the biennial cycle. The biennial cycle was found to exist over a range of parameter values and was not restricted to narrow parameter choices. It should be noted that there are other regions of the parameter space that had a poorer fit to the data that did not exhibit this period 2 oscillation but instead had peaks of equal size as has been observed in some other models [13], [14].

### Parameter variation

We explored differences in the fit statistic and transmission parameter values through the variation of duration of the latency and infectious periods, and the scaling parameters for reduced susceptibility and infectiousness in the older age class. A summary of the results is given in Table 1. We did not investigate the variation in the parameter 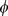 because this simply represents the horizontal fitting of the model to the dataset and does not change the dynamics of the system overall.

**Table 1:** Sensitivity analysis of the fit statistic and fitted transmission parameters for varying duration of immunity and infectious period.

Test conducted	Parameter variation		Fitting parameters			Fit statistic (F)
	Parameter	Value tested	β_0_	β_1_	ν	
Parameters for best fit: α = 0.65; δ = 0.65; γ = 1/1.4; σ = 1/0.57	-	-	1.9896	0.6495	0.0438	89.5751
Reduction in infectiousness for adults	α	0.5	2.5639	0.6775	0.0454	90.3653
	α	0.8	1.6300	0.6262	0.0436	89.6733
Reduction in susceptibility for adults	δ	0.5	2.5534	0.6279	0.0445	90.1470
	δ	0.8	1.6488	0.6667	0.0468	90.2240
Variation to latency period	1/σ	1/0.28	1.9113	0.5879	0.0474	90.0338
	1/σ	1/0.86	2.1255	0.6618	0.0457	92.3239
Variation to infectious period	1/γ	1/1.14	2.3617	0.6478	0.0464	91.6664
	1/γ	1/1.57	1.8289	0.6599	0.0428	90.2565

The assumed values of the parameters are α = 0.65; δ = 0.65; **γ** = 1/1.4; and σ = 1/0.57 unless otherwise varied, as indicated in the table.

Based on the available literature, our initial estimate of the latency period was 4 days. To allow for variation around this value, we tested values within the range of 2 days (weekly rate of 1/0.28) and 6 days (weekly rate of 1/0.86) All values within this range produced a good model fit (F = 90.03 to F = 92.32; Table 1), with the best fit agreeing with our initial estimate of 4 days.

Similar to other RSV models, our model initially assumed the infectious period to be 10 days. This original estimate was based on a study reporting the mean duration of shedding of RSV to be 6.7 days with the range of 1–21 days [23]. An additional study found the shedding duration to be between 3.4 and 7.4 days but considerably longer (9 days and potentially longer) for children aged less than 2 years [28]. While we do not have available viral shedding data, we explored hospital length of stay as a proxy for the infectious period. This assumes that infectiousness is linked to symptom presentation which may not be true. In addition, patients may be discharged from hospital whilst still showing symptoms that are not serious enough to warrant continued hospitalisation. It is expected that hospital stay would be an underestimation of infectious period. From linking the RSV laboratory data to hospitalisation data, we identified 2,424 hospitalisations in children aged less than 2 years in metropolitan Western Australia. The median length of hospital stay was 3 days with the range of 0–6 days. To allow for additional time before and after the hospital stay, where a child may still be infectious, we explored models with infectious periods of 7 days (weekly rate of 1/1) to 12 days (weekly rate of 1/1.7). Despite the epidemiology of RSV infections in Western Australia suggesting the optimal estimate for the infectious period in the vicinity of 5–12 days, only periods in the range of 8–11 days were successful in producing a model with the biennial seasonal pattern; outside this range the model gave an annual pattern which does not concord with the data.

Very few studies have been undertaken to ascertain to what level RSV is transmitted from adults to children, with RSV in adults most often presenting as the “common cold” [24]. One study by Henderson et al (1979) found that the attack rate for first RSV infection was 98%, 75% for second infections and 65% for third infections [25]. As such, values between 0.5 and 0.8 were tested for the scaling parameters α and δ. Suitable model fits, close to the optimal fit, were found for all tests in this range, with the best fits for values between 0.6 and 0.7.

For the parameter ranges tested, the fitted duration of immunity had little variation with typical values around 160 days (ν = 0.0438) and a range from 148 to 164 days. As expected, the most sensitive fit parameter was the average transmission parameter β_0_. Reducing either the susceptibility or infectiousness for the older age class required an increase in the transmission rate for the model to fit the data. Increasing the infectious period reduced the required level of transmission. Varying the latency period did not have a significant impact on the transmission parameter. The fitted value of the seasonality parameter, β_1,_ was consistent across the sensitivity analysis with a value typically around 0.65 and a range from 0.59 to 0.68. The only other model that has been fitted to biennial pattern RSV data assumed an overly large seasonal forcing parameter of β_1_ = 1 and required other parameters to vary season by season to obtain a good fit to the data [15].

The best fitting transmission estimates and the corresponding fit statistic for the different combinations of the duration of immunity and recovery period are shown in Table 1. The fit statistic for realistic parameter choices ranged from F = 89.575 (the best fit) to 92.324. A number of parameter choices produced seasonal epidemics curves of alternating peak sizes, consistent with the RSV data. However, the model that minimised the residual sum of errors assumed a latency period of 4 days and infectious period of 10 days, in keeping with earlier estimates in the literature.

## Discussion

RSV is the most commonly detected pathogen in children with ALRI and both RSV testing and positive detections in Western Australia exhibit distinct seasonality with alternating peaks in winter months. Using positive RSV detections obtained through population-based data linkage, we have constructed a simple mathematical model to mimic these seasonal patterns of detections in the metropolitan population of Western Australia. Our age-structured model accurately captures the biennial seasonal epidemic curves that were observed in children aged less than 2 years between 2000 and 2005. The compartmental SEIRS model, based on several assumptions, now provides a base model on which to expand and increase complexity.

All models are based on assumptions of epidemiological parameters of the disease in question. There is a paucity in the literature of the characteristics of RSV infection including duration of shedding and infectiousness. Previous models have used the infectious period of 10 days based on a study of 23 infants [23]. In another Kenyan community study of 193 children, the mean duration of shedding was 4.5 days and varied between severity of infection and recent history of infection [29]. While we investigated models with infectious periods ranging from 7 to 12 days, we could only replicate the biennial seasonal peaks of RSV with infectious periods between 8 and 11 days; outside this range a suitable fit could not be found with only annual patterns produced by the model.

Our initial estimate for the duration of immunity was 200 days (∼6.6 months), based on previous RSV models in the literature [13]. We investigated this estimate using data regarding RSV reinfections. From our linked laboratory data, there were 4,920 RSV positive identifications between 2000 and 2005 throughout Western Australia in those aged less than 2 years. Of these, 92.6% were single infections and 363 (7.4%) were reinfections (6.3% were second time infections, 0.7% third time infections and 0.2% fourth time infections). Excluding those reinfections with positive RSV identifications from specimens collected less than 14 days apart (most likely to be indicative of the same infection), there were 99 reinfections where the time between positive RSV identification was >14 days. Of these 99 reinfections, 80 were identified in children from metropolitan Western Australia. The mean time between subsequent RSV infections from reinfections in metropolitan children was 250 days (8.21 months) and the range was 15–592 days. However, the duration of immunity that the produced the best fit for the model was 160 days. It is possible that immunity wanes several months before the onset of a subsequent RSV season, but that there are not enough infected individuals in the population at that time for the infection to gain momentum and hence our estimate of the immunity period from the data is an overestimate.

We have identified several improvements that can be made to the model which will become the focus of future work. First, we will improve the age structure of the model to allow the transmission parameters to vary between the different age groups as opposed to our current model where we assume the same transmission rate for each age group with a fixed scaling parameter. The incidence of RSV is greater in infants aged less than 12 months than those in the second year of life, as we have previously shown with hospitalisation rates for bronchiolitis for which RSV is mostly associated [19]. Therefore a natural next step is to further refine the age classes into those aged less than 12 months, 12–23 months and 2 years and over. This will result in a higher number of parameters to fit to the data and therefore will increase the complexity of the model. The advantage is that age-specific interventions, such as vaccination, are easily incorporated in the modelling framework.

Second, we will introduce non-metropolitan data on RSV detections and investigate the changes in the parameters. Parts of rural and remote Western Australia experience tropical climate and therefore, are very likely to experience a different seasonal pattern in terms of peak height and duration. Our total population-based data allows us to investigate seasonality based on geographical location. To achieve this accurately, we will investigate whether the transmission parameters can be modelled as functions of climatic variables of each geographical region such as average weekly temperatures, rainfall and humidity. A study in Utah correlated climatic variables to hospitalisation data coded for RSV and RSV-bronchiolitis and concluded that temperature and wind speed were the best fitting climatic variables [30]. Due to the differing geographical regions, the effect of corresponding climatic variables on RSV detections may be different in Australia. Indeed, other RSV transmissions with an annual pattern have had lower estimates for the seasonal forcing parameter β_1_, depending on the climatic region. In models from tropical regions, β_1_ has been typically small (e.g. Gambia: 0.16–0.20; Florida: 0.10–0.13 and Singapore: 0.14–0.20); whereas models from temperate climate regions with more distinct seasonality have a larger β_1_ (e.g. Finland: 0.16–0.39) [13].

Third, to measure the likely impact of future interventions, we will model the introduction of a newborn vaccination program and predict the effect on RSV incidence. Such analyses have been previously attempted by modelling [14] and economic models using Monte Carlo simulation methods [31]. However, until such time as accurate vaccine efficacy estimates can be derived from vaccine trial data, these models will continue to be based on multiple assumptions. The benefit of this modelling approach is that different scenarios around age of vaccination, efficacy and waning period of the vaccine can be investigated to inform future vaccination policy.

We also did not investigate RSV transmission by Aboriginality or other risk groups for RSV. Our previous analysis of metropolitan RSV identifications showed no difference in seasonality of RSV in metropolitan Aboriginal and non-Aboriginal children [3]. However, as we extend our model to include non-metropolitan data, disaggregating according to Aboriginality will be important due to the higher burden of illness in Aboriginal children from rural and remote areas [32], [33].

## Conclusions

We have successfully developed a model to mimic the biennial seasonal epidemic curves of RSV identifications in metropolitan Western Australia. A strength of our model is the quality of data that it is based on, gathered from individual-level linked total population-based data sources. Not all RSV positive detections are associated with hospitalisations so it is important to not limit data sources to the severe end of the clinical spectrum. RSV continues to be a major pathogen in children and until such time that positive identifications of RSV become mandatory, future studies will need to rely on routinely collected laboratory data.
